# Body composition in patients with Fontan physiology: a systematic review

**DOI:** 10.1007/s00431-023-05100-2

**Published:** 2023-08-05

**Authors:** Rubens J. van den Berg, Jayanti N. Pos, Linda E. Scheffers, Linda E. M. van den Berg, Willem A. Helbing

**Affiliations:** 1 Department of Pediatrics, Division of Pediatric Cardiology, Erasmus MC-Sophia Children’s Hospital, Doctor Molewaterplein 40, 3015 GD Rotterdam, The Netherlands; 2 Department of Orthopedics and Sports Medicine, Erasmus MC-Sophia Children’s Hospital, Rotterdam, The Netherlands; 3 Department of Radiology, Erasmus MC-Sophia Children’s Hospital, Rotterdam, The Netherlands

**Keywords:** Fontan procedure, Body composition, Muscle mass, Fat mass, Single ventricle

## Abstract

Fontan circulation is a highly abnormal circulatory state that may affect various organ systems. The effect on body composition is an important factor to assess the condition of the patient. This systematic review assesses body composition and possibly related adverse outcomes in patients with a Fontan circulation, to provide an overview of current insights. Studies evaluating body composition by compartment (either fat mass or lean/muscle mass) in Fontan patients published up to April 2023 were included in this systematic review. Of 1392 potential studies, 18 studies met the inclusion criteria. In total, body composition measurements of 774 Fontan patients were included. Body composition was measured using dual-energy X-ray absorptiometry (DXA) (*n* = 12), bioelectrical impedance analysis (BIA) (*n* = 5), computer tomography (CT) (*n* = 1), or magnetic resonance imaging (MRI) (*n* = 1). All studies reported a normal body mass index (BMI) in Fontan patients, compared to controls. Five out of nine studies reported significantly higher body fat values, and twelve out of fifteen studies reported significantly lower muscle or lean mass values in the Fontan population compared to the healthy population. Unfavorable body composition in Fontan patients was associated with decreased exercise capacity, worse cardiac function, and adverse outcomes including hospital admissions and death.

*   Conclusions*: Despite having a normal BMI, Fontan patients have an increased fat mass and decreased muscle mass or lean mass compared to the healthy population. This unfavorable body composition was associated with various adverse outcomes, including a decreased exercise capacity and worse cardiac function.

**Table:** 

**What is Known:** *• Patients with a Fontan circulation have a decreased exercise capacity compared to healthy peers, an unfavorable body composition might be a contributor to their impaired exercise capacity.*	
**What is New:** *• Fontan patients are predisposed to an unfavorable body composition, characterized by increased fat mass and decreased muscle mass accompanied by a normal BMI compared to the healthy population.* *• Among others, unfavorable body composition was associated with decreased exercise capacity, cardiac function, and increased morbidity in patients with a Fontan circulation.*

**Supplementary Information:**

The online version contains supplementary material available at 10.1007/s00431-023-05100-2

## Introduction

The Fontan procedure is the preferred treatment strategy in children born with a functional univentricular heart, unsuitable for biventricular repair [1]. Although long-term survival after the Fontan procedure has been improving over the years, patients are still facing increased morbidity and mortality over time [2]. While infancy patients with a single ventricle experience growth impairment, adult Fontan patients show high rates of overweight and obesity [3, 4]. Adiposity is often expressed as body mass index (BMI) [3]. However, when BMI is used as a surrogate of adiposity, it does not take into account the patient’s body fat versus muscle mass. Therefore, the combination of reduced muscle mass and increased body fat may result in “normal” BMI and thereby conceal the presence of increased adiposity [5]. A more accurate way to approximate body composition is by compartment: fat free mass (consisting out of lean mass and bones) and fat mass. As total body lean mass still includes organs (which could be enlarged in Fontan patients), leg lean mass is most likely the best approximation for skeletal muscle, as most muscles are located in the legs. Several studies showed that estimating body composition by compartment by using dual-energy X-ray absorptiometry (DXA) is a better predictor of cardiovascular risk factors and all-cause mortality than just BMI [6–8]. Gaining more knowledge on the body composition of Fontan patients is important, as it might be a contributor to their impaired exercise capacity [9]. Having sufficient muscle mass might be especially important in patients with a Fontan circulation [10, 11]. In Fontan patients, the systemic venous return bypasses a subpulmonary ventricle and is directly connected to the pulmonary arteries [12]. Due to the absence of a subpulmonary ventricle to pump blood through the pulmonary vascular bed, cardiac filling is more dependent on the respiratory and peripheral muscle pumps [10]. To the best of our knowledge, no review regarding body composition in patients with a Fontan circulation exists in current literature. This systematic review is aimed at (1) assessing body composition by compartment in patients with a Fontan circulation and (2) investigating the prognostic value of (an unfavorable) body composition in this population.

## Methods

### Protocol registration and search strategy

This study was conducted according to the PRISMA guidelines [13]. A systematic search was performed in online databases on 13 April 2023. The search is presented in Supplement 1. The searched databases were Embase, Medline Ovid, Web of Science, and Cochrane CENTRAL. The search terms consisted of keywords related to Fontan circulation and body composition values. No filters were applied.

### Inclusion and exclusion criteria

All studies were screened on title and abstract by two independent reviewers (JNP and RJB). Studies were included when they described body composition measurements by compartment in patients with a Fontan circulation. The full text of relevant articles was assessed for inclusion in the systematic review. The exclusion criteria were as follows: (a) studies without patients with a Fontan circulation, (b) body composition values of interest (body fat percentage, fat mass (index), lean mass (index), appendicular lean mass (index), or leg lean mass (index)) were not mentioned, (c) body composition values not specifically matched to patients with a Fontan circulation, and (d) results from conference papers and abstracts without a full text available. Discrepancies between the reviewers were discussed and resolved together with a third reviewer (LES).

### Data extraction

From the resulting articles, two reviewers (JNP and RJB) extracted all data needed independently using Excel (version 2019). The extracted data from each reviewer was verified by the extracted data from the other reviewer; any discrepancies were discussed together with a third author (LES). The following data were extracted: study characteristics—journal, year of publication, study design, study size (of the patient group and, if present, of the control group), time frame of inclusion, and location or database; patient characteristics—age, age at Fontan completion, sex, Fontan type, ventricle type, and the method used to compare the body composition of the patients with a Fontan circulation (control group or pre-existing reference values); methodology used for measuring the body composition; measurement outcomes—body mass index (BMI), body fat percentage, appendicular lean mass (index), leg lean mass (index), fat mass (index), and lean mass (index); conclusions of studies related to the relationship between body composition and (adverse) outcomes (such as re-interventions and cardiac failure); and conclusions related to the body composition measurements. Studies were divided into groups and summarized based on the age of the study population—adults only, children only, and both adults and children.

### Quality assessment appraisal

The Strengthening Reporting of Observational Studies in Epidemiology (STROBE) checklist was used to assess the quality of cohort and cross-sectional studies [14].

## Results

### Included studies

**Fig. 1:**
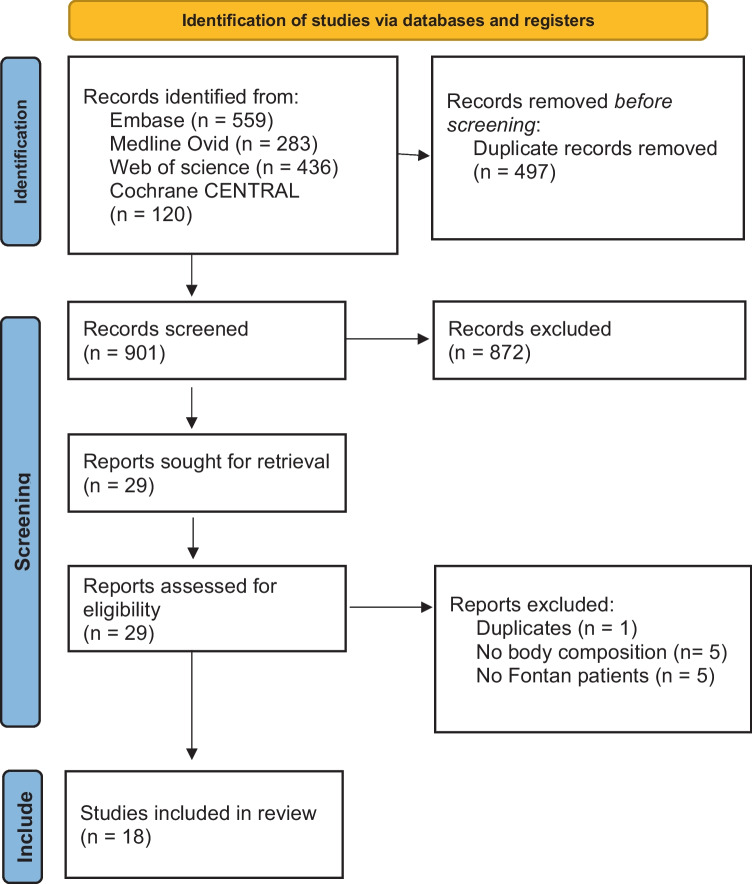
Flowchart study inclusion

A total of 1392 studies were identified through the search. After removing duplicates and screening on title and abstract, the full text of 29 articles was screened. A total of 18 studies were included in this systematic review [5, 15–31]. This process is presented in Fig. 1.

### Quality appraisal

None of the studies were excluded based on quality. All studies met over 75% of the STROBE checklist criteria. The quality assessment is presented in Supplement 2.

### Study and patient characteristics

The study designs of all included studies are described in Table 1, and patient characteristics in Table 2. In total, body composition was measured in 774 patients with a Fontan circulation, and 298 healthy participants served as controls. Five studies included children only [15, 16, 24, 27, 28], five studies included adults only [5, 17, 18, 25, 32], and eight studies included both children and adults [19–23, 26, 30, 31]. The mean age of the patients between studies ranged from 1.5 to 30 years; 45.0% of the participants was female. The mean age at Fontan completion was 4.44 years. The percentage of a dominant left ventricle was 52.6%, and the percentage of patients with an open fenestration was 49.8%.

**Table 1:** Study characteristics

Studies	Location or database	Journal	Year of publication	Study design	Measurement method	Time frame of inclusion
**Studies including children only:**						
Avitabile et al. [27]	Children’s Hospital of Philadelphia and Cincinnati Children’s Hospital Medical Center	Journal of American Heart Association	2022	Cohort	DXA	January 2019–November 2021
Pyykknönen et al. [28]	Helsinki University Hospital	Pediatric Cardiology	2022	Prospective interventional study	BIA	–
Hansson et al. [15]	Northern part of Sweden and Stockholm	Cambridge University Press	2021	Cross-sectional	DXA	2010–2019
Sandberg et al. [16]	Northern part of Sweden and Stockholm	Cardiology in the Young	2020	Cross-sectional	DXA	September 2017–October 2018
Sarafoglou et al. [24]	Children’s Hospital and Clinics of Minnesota and the Masonic Children’s Hospital at the University of Minnesota, Minneapolis, Minnesota, United States of America	Cardiology in the Young	2020	Cross-sectional	DXA & CT	–
**Studies including adults only:**						
Chemello et al. [32]	University Hospital of Padua	Journal of Cardiovascular Development and Disease	2021	Cohort	BIA	May 2017–May 2020
Tran et al. [5]	Adult Congenital Heart Disease Database at Royal Prince Alfred Hospital and Australian and New Zealand Fontan Registry (ANZFR)	Journal of the American Heart Association	2020	Cross-sectional	DXA	March 2016–February 2018
Ohuchi et al. [17]	–	International Journal of Cardiology	2019	Cohort	DXA	September 1990–Septermber 2017
Shiina et al.[25]	St. Luke’s International Hospital	Congenital Heart Disease	2018	Cohort	BIA	July 2016–December 2016
Cordina et al. [18]	Coronary Heart Disease Database at Royal Prince Alfred Hospital (RPAH), Sydney, Australia	Heart–BMJ Journals	2013	Cohort	BIA	–
**Studies including children and adults:**						
Tekerlek et al. [30]	–	Elsevier	2023	Case–control	BIA	September 2021–January 2022
Wadey et al. [31]	Adult Congenital Heart Disease Database at Royal Prince Alfred Hospital and Australian and New Zealand Fontan Registry (ANZFR)	Journal of American Heart Association	2022	Cohort	DXA	March 2016–February 2018
Cao et al. [19]	Australian and New Zealand Fontan Registry (ANZFR)	International Journal of Cardiology	2021	Cohort	DXA	–
Vaikunth et al. [22]	Children’s Hospital of Philadelphia	The Journal of Pediatrics	2021	Cohort	DXA	July 2011–October 2013
Possner et al. [26]	Cincinnati Children’s Hospital Medical Center	CJC Open	2020	Cross-sectional	DXA	2000–2018
Powell et al. [23]	Cincinnati Children’s Hospital	Journal of the American Heart Association	2020	Cohort	Liver or abdominal MRI	April 2019–January 2020
Avitabile et al. [20]	Children’s Hospital of Philadelphia	Heart–BMJ Journals	2018	Cohort	DXA	July 2011–October 2013
Avitabile et al. [21]	Children’s Hospital of Philadelphia	Heart–BMJ Journals	2014	Cohort	DXA	July 2011–October 2013

*–* not reported, *DXA* Dual-energy X-ray Absorptiometry, *BIA* Bioelectrical Impedance Analysis, *CT* Computed Tomography, *BMJ* British Medical Journal

**Table 2:** Patient characteristics

Studies	Patient group (*n*)	Control group (*n*)	Age (years)	Age at Fontan completion (years)	Female (%)	Fontan type (*n*)	Dominant left ventricle (%)	Open fenestration (%)
**Studies including children only:**								
Avitabile et al. [27]	20	–	15.6 ± 1.7	–	50%	ECC 14, LT 6	45%	95%
Pyykkönen et al. [28]	16	–	14.5 ± 2.6	2.9 ± 0.5	37.5%	–	31.3%	6.3%
Hansson et al. [15]	38	38	12.3 ± 3.9	2.4 ± 0.9	54%	–	39%	–
Sandberg et al. [16]	43	43	12.2 ± 3.9	–	44%	–	56%	–
Sarafoglou et al. [24]	10	11	12.2 ± 1.69	–	30%	–	30%	–
**Studies including adults only:**								
Chemello et al. [32]	43	–	30 ± 9	–	39.5%	ECC 20, LT 19, unknown 4	74.4%	–
Tran et al. [5]	28	–	26 ± 7	–	54%	APC 3, ECC 14, ICC 11	57%	32%
Ohuchi et al. [17]	97	–	22.3 ± 5	–	36%	APC 7, ECC 47, ICC 43	42%	–
Shiina et al. [25]	46 (13 Fontan patients)	12	29 ± 5.9	–	54%	ECC 11, LT 2	–	–
Cordina et al. [18]	16	8	30 (SEM 2)	–	19%	APC 6, TCPC 10	50%	–
**Studies including children and adults:**								
Tekerlek et al. [30]	21	21	16.4 ± 6.6	9.6 ± 4.69	23.8%	–	66.7%	47.6%
Wadey et al. [31]	89	–	23.3 ± 6.7	–	52.3%	APC 12, 23 LT, 54 ECC	61%	–
Cao et al. [19]	144	–	23 ± 8 (13–45)	5.1 ± 6	46%	APC 19, ECC 87, LT 35, unknown 3	59%	28%
Vaikunth et al. [22]	46	–	12.5 ± 5.3 (5.1–20)	2.8 (1.4–15.7)	50%	ECC 27, LT 19	43%	83%
Possner et al. [26]	40	–	25.5 ± 7.9	4.4 ± 3.8	50%	APC 7, ECC 11, LT 2	68%	–
Powell et al. [23]	47	165	15 ± 3.1	3.9 ± 1.9	47%	ECC 31, LT 14, unknown 2	–	–
Avitabile et al. [20]	13	–	17.7 (12.7–26)	–	46%	ECC 5, LT 8	54%	69%
Avitabile et al. [21]	50	–	11.5 (5.1–33.5)	–	48%	ECC 29, LT 21	32%	84%

*–* not reported, *SD* standard deviation, *SEM* standard error mean, *APC* atriopulmonary connection, *ECC* extracardiac conduit, *ICC* intracardiac conduit, *LT* lateral tunnel Fontan, *TCPC* total cavopulmonary connection

Outcomes were given in mean ± SD or median (range)

### Measurement methods

The measurement methods are summarized in Table 1. Twelve studies assessed the body composition using dual-energy X-ray absorptiometry (DXA) [5, 15, 16, 18–22, 27, 28, 30–32]. Five studies estimated the body composition using bioelectrical impedance analysis (BIA) [17, 23, 25, 28, 32]. One study assessed the body composition using peripheral quantitative computed tomography (CT) taken at a specific location on the left tibia, corresponding to 66% site of its total length [24]. One study assessed the body composition using liver or abdominal magnetic resonance (MR) at levels thoracic vertebrae 12 (T12) and lumbar vertebrae 3 (L3) [26]. Seven studies recruited control patients [15, 16, 18, 23–25, 30]. Nine studies used reference values to compare their patient group [5, 19–22, 24, 27, 31]. Two studies did not compare the body composition with control patients or reference values [17, 32]. Eight out of eighteen studies [5, 18–21, 23, 27] used *Z*-scores. The studies indexed their results on sex and age [5, 18, 19], sex, age, race, height, BMI, and leg-length [20–22, 27] or did not mention how they normalized their results [23].

### Body composition

Out of the 18 included studies, 17 calculated the BMI of Fontan patients and controls or reference values [5, 15, 16, 18–28, 30–32]. These outcomes are documented in Table 3. Nine studies measured fat mass in patients with a Fontan circulation [5, 15, 17, 19, 21, 23, 25, 30, 32], and 15 measured lean/muscle mass in patients with a Fontan circulation [5, 15, 16, 18–28, 31]. Outcomes regarding fat, lean, and muscle mass can also be found in Table 3. All measurement outcomes are summarized in Table 4. Measured BMI was within normal range and not significantly different from control patients or reference values in fifteen out of seventeen studies [5, 15, 16, 18–28, 30–32]. Five out of nine studies concluded that patients with a Fontan circulation had a higher fat mass [5, 15, 19, 23, 31]. Four studies concluded that Fontan patients had a comparable lean/muscle mass to controls/reference values [16, 24, 26, 30]. Twelve out of fifteen studies concluded that patients with a Fontan circulation had a reduced (skeletal) muscle mass and/or lower lean mass [5, 15, 18–23, 25–27, 31].

**Table 3:** Measurement method body composition and outcome BMI

Studies	BMI patients (kg/m^2^)	Appendicular lean mass (index)	Leg lean mass (index)	Lean mass (index)	Fat mass (index)	Body fat (%)	Conclusion
**Studies including children**							
Avitabile et al. [27]*	*Z*-score: − 0.08 ± 1.01	–	*Z*-score: − 1.38 ± 1.02	–	–	–	Fontan patients had normal BMI and lower leg lean mass compared to reference values
Pyykkönen [28]	21 ± 3	–	0.3 ± 0.06 kg/kg	–	–	–	Fontan patients had normal BMI and fat and lean muscle mass in the normal range
Hansson et al. [15]	19.3 ± 3.7; *Z*-score: 0.22 ± 1.2 *Controls:* 18.5 ± 3.3; *Z*-score: − 0.13 ± 1.0	–	4.01 kg/m^2^ ± 0.9 kg/m^2^ *Controls*: 4.4 kg/m^2^ ± 1.1 kg/m^2^	Total body: 12.90 ± 2.0; kg/m^2^ Arms: 1.26 ± 0.4; kg/m^2^ Abdomen: 6.30 ± 1.0 kg/m^2^ *Controls*: Total body: 13.28 ± 2.4; kg/m^2^ Arms: 1.36 ± 0.4; kg/m^2^ Abdomen: 6.29 ± 1.1 kg/m^2^	Total body: 5.75 ± 2.2; kg/m^2^ Arms & legs: 0.65 ± 0.2 kg/m^2^ & 2.05 ± 0.6; kg/m^2^ Abdomen: 2.70 ± 1.4 kg/m^2^ *Controls*: Total body: 4.4 ± 1.8; kg/m^2^ Arms & legs: 0.53 ± 0.2 kg/m^2^ & 1.85 ± 0.7; kg/m^2^ Abdomen: 1.68 ± 0.9 kg/m^2^	–	Fontan patients had normal BMI, lower lean mass, and higher fat mass compared to controls
Sandberg et al. [16]	18.9 ± 3.6 *Z*-score: 0.1 ± 1.1	–	4.0 kg/m^2^ ± 0.9 *Controls*: 4.2 kg/m^2^ ± 1.0	–	–	–	Fontan patients had normal BMI and lower leg lean mass compared to controls
Sarafoglou et al. [24]^a^	18.4 ± 2.98 *Controls:* 17.4 ± 1.67	–	Tibial muscle density: 82.64 mg/cm^3^ ± 1.59 mg/cm^3^ *Controls*: Tibial muscle density: 83.51 ± 1.20 mg/cm^3^	–	–	–	Fontan patients had normal BMI and comparable muscle mass in the tibia compared to controls. Indexing was not performed in this study
**Studies including adults:**							
Chemello et al. [32]	Men: 22.2 ± 2.8 Women: 21.8 ± 4.3					Men: 18.5 ± 5.7 Women: 31.7 ± 8.2	BMI fell within normal range, no comparison to reference values was made for body composition outcomes
Tran et al. [5]**	22.4 (IQR: 20.4–27.3) *Z*-score: 0.38 ± 1.32	18.0 kg ± 4.3; 6.4 ± 1.0 kg/m^2^ *Z*-score: − 1.49 ± 1.10 Trunk/appendicular fat ratio: 1.3 (IQR: 1.1–1.8)	–	15 kg/m^2^ (IQR: 13.9–16.2) *Z*-score: − 0.7 ± 1.15	6.5 kg/m^2^ (IQR: 4–9.9)	30 ± 11 *Z*-score: 0.23 ± 1.26	Fontan patients had normal BMI, lower skeletal muscle mass, and increased adiposity compared to reference values
Ohuchi et al. [17] *n* = 74/*n* = 23	–	–	–	–	Subgroup ↑ Peak VO2 over time: 20% ± 8 Subgroup ↓ Peak VO2 over time: 27% ± 8	–	Body fat percentage was lower, and the fat-free percentage was higher in Fontan patients that improved on Peak VO_2_ over time
Shiina et al. [25]	19.7 ± 4.7 *Controls:* 21.6 ± 2.1	–	–	Skeletal muscle: 34.7 ± 4.4 kg/m^2^ *Controls*: Skeletal muscle: 43.8 ± 4.4 kg/m^2^	24% ± 9.9 *Controls*: 20.1% ± 4.0	–	Fontan patients had normal BMI, lower skeletal muscle mass, and comparable fat percentage compared to controls
Cordina et al. [18]**	25.5 (SEM 0.8) *Controls:* 26 (SEM 3)	–	–	Total body: 16.5 kg/m^2^ (SEM 0.4)	7.1 kg/m^2^ (SEM 0.2) *Z*-score: − 1.46 (SEM 0.22)	–	Fontan patients had normal BMI, reduced lean mass, and appendicular lean mass compared to controls
**Studies including children and adults:**							
Tekerlek et al. [30]	18.7 ± 3.9 kg/cm^2^ *Controls*: 20.89 kg/cm^2^ ± 3.7 kg/cm^2^	–				14.4 ± 7.5 *Controls*: 17.1 ± 7.6	Fontan patients had lower BMI and comparable fat ratio compared to controls
Wadey et al. [31]	23.7 ± 5.0	17.8 ± 4.5 kg	–	42.7 ± 9.2 kg	–	–	Fontan patients had decreased lean body mass and increased body fat. Indexing was not performed in this study
Cao et a. [19]**	23 ± 4.7	*Z*-score: − 1.4 ± 1.1	–	–	29% ± 10 *Z*-score: 0.3 ± 1.1	*Z*-score: 0.1 ± 1.3	Fontan patients had normal BMI; 1/3 had reduced skeletal muscle mass and normal fat mass compared to reference values
Vaikunth et al. [22]*	*Z*-score: 0.09 ± 0.97 (*R*: − 2.49–2.24)	–	*Z*-score: − 0.9 ± 1.1 (*p* < 0.001)	–	–	–	Fontan patients had normal BMI and reduced leg lean mass compared to controls
Possner et al. [26]^b^	25.2 ± 4.6	–	–	Skeletal muscle mass T12: 10.5 ± 2.5 cm^2^/m^2^ Skeletal muscle mass L3: 50.7 ± 8.1 cm^2^/m^2^ *Controls:* Skeletal muscle mass^1^ of males L3: 60.9 ± 7.8 cm^2^/m^2^	–	–	Fontan patients had normal BMI and reduced skeletal muscle mass measured at L3 in male Fontan patients compared with healthy controls
Powell et al. [23]***	22.1 ± 5.7 *Z*-score: 0.05 ± 1.4 *Controls:* 21.8 ± 4.1	–	Right leg: 6.0 ± 2.1; kg *Z*-score: − 0.5 ± 1.0 Left leg: 6.0 ± 2.1; kg *Z*-score: − 0.5 ± 1.0 *Controls:* Right leg: 7.1 ± 2.1; kg Left leg: 7.1 ± 2.1 kg	Right arm: 2.0 ± 0.8; kg *Z*-score: − 0.4 ± 0.9 Left arm: 2.0 ± 0.8; kg *Z*-score: − 0.4 ± 1.0 Trunk: 18.3 ± 5.4 kg *Z*-score: − 0.4 ± 1.0 *Controls:* Right arm: 2.4 ± 0.9; kg Left arm: 2.3 ± 0.8; kg Trunk: 20.5 ± 5.3 kg	26.0% ± 11.8 *Z*-score: 0.4 ± 1.2 *Controls:* 22.4% ± 9.6	–	Fontan patients had normal BMI, lower lean body mass, and skeletal muscle mass and higher fat percentage compared to controls
Avitabile et al. [20]*	–	–	*Z*-score: − 0.87 ± 0.95 (*R*: − 2.53–1.11)	–	–	–	Fontan patients had lower leg lean mass compared to controls
Avitabile et al. [21]*	*Z*-score: 0.15 ± 0.98 (*R*: − 2.49–2.24)	–	*Z*-score: − 0.89 ± 0.91	*Z*-score: − 0.33 ± 0.77	–	*Z*-score: 0.22 ± 0.99	Fontan patients had normal BMI and lower leg lean mass compared to reference values

*–* not reported, *KG* kilograms, *M* meters, *R* range, *IQR* interquartile range, *SEM* standard error of mean

^a^Sarafoglou et al. measured cross-sectional area instead of lean mass

^b^Possner et al. measured skeletal muscle mass instead of lean mass

*Growth and body composition variables were converted to *Z*-scores (SD scores). The 2000 Centers for Disease Control and Prevention growth charts were used to calculate sex-specific *Z*-scores for height, weight, and body mass index relative to age; the leg lean and fat mass *Z*-scores were adjusted for leg length; **Z-scores for body composition are adjusted for sex and age; **Z-scores for body composition did not mention how they normalized their *Z*-scores

**Table 4:** Summary outcomes body composition compared to controls or reference values

Studies	Comparison	Measurement outcomes		
		BMI	Fat mass	Muscle mass
**Children only**				
Avitabile et al. [27]	Reference	Normal	Na	Decreased
Pyykkönen et al. [28]	No comparison	No comparison	Na	No comparison
Hansson et al. [15]	Controls	Normal	Higher	Decreased
Sandberg et al. [16]	Controls	Normal	Na	Comparable
Sarafoglou et al. [24]	Reference	Normal	Comparable	Comparable
**Adults only**				
Chemello et al. [32]	No comparison	No comparison	No comparison	Na
Tran et al. [5]	Reference	Normal	Higher	Decreased
Ohuchi et al. [17]	No comparison	Na	No comparison	Na
Shiina et al. [25]	Controls	Normal	Comparable	Decreased
Cordina et al. [18]	Controls	Normal	Na	Decreased
**Children and adults**				
Tekerlek et al. [30]	Controls	Normal	Na	Comparable
Wadey et al. [31]	Reference	Normal	Higher	Decreased
Cao et al. [19]	Reference	Normal	Comparable and higher	Decreased
Vaikunth et al. [22]	Reference	Normal	Na	Decreased
Possner et al. [26]	Reference	Normal	Na	Decreased (in males), comparable (in females)
Powell et al. [23]	Controls	Normal	Higher	Decreased
Avitabile et al. [20]	Reference	Na	Na	Decreased
Avitabile et al. [21]	Reference	Normal	Comparable	Decreased

Na = not applicable (if outcomes were not reported in the article)

### Correlations between body composition and (adverse) outcomes

Five studies investigated associations between body composition and cardiac function [5, 19, 20, 23, 25]. Tran et al. showed that the degree of muscle deficit is associated with ventricular systolic impairment [5]. This corresponds to the findings of the other four studies. A higher leg lean mass was associated with larger increases of cardiac index and indexed systemic flow during exercise [20]. Higher body fat was associated with poorer New York Heart Association class (III or IV) in study of Shiina et al. [25]. Also, various Fontan complications including cardiac failure (New York Heart Association class III/IV), protein-losing enteropathy, plastic bronchitis, heart transplant, and death were associated with increased adiposity and/or lower muscle mass in the studies of Cao et al. and Powell et al. [19, 23]. Cao et al. demonstrated that every 1% increase in body fat percentage was associated with a 10% increased risk of reaching a Fontan complication. In total, eight studies investigated the relation between body composition and exercise capacity [5, 17, 18, 20, 23, 26]. Higher muscle mass was correlated with higher exercise capacity (Peak VO2) in three studies; higher leg lean mass showed the same association [5, 18, 23, 26]. Avitabile et al. found a correlation between skeletal muscle deficits and a reduced exercise capacity [20]. Patients with an improving exercise capacity over time had a lower body fat percentage compared to patients with a Fontan circulation that diminished in exercise capacity over time [17]. Lastly, Vaikunth et al. also showed that lower leg lean mass was associated with a lower whole body bone mass density [22]. Two studies investigated the effect of exercise training on (leg) lean muscle mass, both showing no change [27, 29].

## Discussion

This is the first systematic review to assess the body composition in patients with a Fontan circulation. Overall, studies examining body composition in patients with a Fontan circulation were scarce. A total of 18 studies including 774 patients with a Fontan circulation met the inclusion criteria. All studies reported normal BMI, in contrast to body composition outcomes. The majority of the studies reported a decreased measured muscle mass and increased fat mass in patients with a Fontan circulation. Multiple studies looked at the relationship between body composition and adverse outcomes, showing that an unfavorable body composition in patients with a Fontan circulation was associated with impaired cardiac function, decreased exercise capacity and increased amount of Fontan complications.

### Body composition measurement methods

The findings of this systematic review are heterogeneous. Reviewed studies included both children and adults, and different techniques were used to measure body composition. Most studies used DXA or BIA to measure the body composition. The use of DXA to measure different body compartments has been described and validated against the gold standard (whole-body potassium counting) in multiple clinical studies, showing comparable outcomes [34]. As DXA scans are easier, cheaper, and less stressful compared to whole-body potassium counting for the patient, DXA is the recommended measuring methods for assessing body composition in both research and clinical setting [34]. However, the use of DXA is limited to the research/ clinical context due to equipment cost, exposure to radiation (although very low), and lack of portability [35]. BIA has been proven to be one of the most practical methods to estimate body composition, as costs are low, its quick assessment procedures, and accessibility [36]. However, measurements obtained with BIA are less accurate and influenced by sex, ethnicity, weight, and age [37]. Also, diseases which change serum electrolytes, blood flow, and fluid distribution affect the accuracy of BIA measurements, ultimately making BIA less accurate for Fontan patients [37]. MRI and CT were used in only two studies [23, 24]. MRI and CT can measure both whole-body composition and body composition of a certain area. A study from Kullberg et al. showed strong correlations between MRI, CT, and DXA; however, both MRI and CT have major disadvantages [38]. MRI measurements might be highly dependent on used scanning protocols and are very expensive and time-consuming. Although CT is much faster, exposure to radiation is high; in addition, both techniques are very costly. Surprisingly, our systematic review did not find any reporting on the use of air-displacement plethysmography or skinfold measurements in patients with a Fontan circulation.

### Body composition outcomes

As mentioned earlier, BMI is the most commonly used measurement to define adiposity [39]. However, BMI can be misleading as a combination of reduced lean mass and increased adiposity can result in a “normal” BMI, as was confirmed by this systematic review. In most included studies, patients with a Fontan circulation had an unfavorable body composition profile characterized by reduced skeletal muscle mass and increased adiposity, despite a normal BMI. The only two studies reporting normal muscle mass compared to the healthy population were performed in children. These findings may have important diagnostic and prognostic implications. In the general population, an unfavorable body composition is associated with various morbidities, including diabetes, cardiovascular disease, cancer, and early death [40–42]. Studies included in this systematic review also found an association between unfavorable body composition in Fontan patients and adverse outcomes, including cardiac failure [19, 23]. This is particularly relevant since several studies have shown the importance of the skeletal muscle pump to increase venous return during exercise [18, 20]. The study by Avitabile et al. showed that having a higher leg lean mass was associated with increases in cardiac index during exercise, suggesting that Fontan patients with higher leg lean mass may indeed be better able to augment systemic output during exercise [20]. In addition, all studies investigating the association between (leg) lean mass and exercise capacity included in this systematic review found that a reduced lean mass was associated with worse exercise capacity [5, 18, 23, 26]. While this may be intuitive, these findings stress the importance of maintaining and improving muscle mass in these patients. Causes of muscle mass deficits are poorly defined in patients with a Fontan circulation. Relative deconditioning, chronically elevated central venous pressure, decreased physical activity levels and neurohormonal activation, and malnutrition might be contributing factors [43–45]. As prospective studies are currently lacking, the question also remains whether worse Fontan outcomes are caused by less physical activity leading to an unfavorable body composition, or the other way around. A way to improve body composition in the healthy population is by performing moderate to vigorous physical activity [46, 47]. Exercise in patients with a Fontan circulation has been shown to improve exercise capacity, cardiac function and quality of life [9]. While there are few existing data supporting any particular approach, establishing a healthy lifestyle in Fontan patients, is likely to have a substantive positive impact. Recent studies from both Avitabile et al. and Chemello et al. measured body composition respectively before and after lower leg-focused muscle exercise and an 6-month lasting exercise intervention; both studies did not find a significant change [27, 29].

### Limitations

Several limitations of the studies included have to be discussed. Patient characteristics were not always reported in detail, and information regarding physical activity levels of the included patients was lacking. The majority of the studies did not include a healthy control group but compared the body composition outcomes of the patients to reference values. Information regarding the exact origin of these reference values and methods used to index mass was often lacking, some studies did not correct their results for height at all which is especially important in studies including children. Some results were indexed very well by certain notable exceptions [20–22, 27]. Two studies did not compare body composition outcomes to their healthy population, making it hard to draw definite conclusions from their data [17, 28]. Body composition outcomes of children and adults (in the mixed studies) and males/females were not reported separately; also cohorts were homogeneous. We could not rule out overlap of patients between studies for all studies included in our review. Hansson et al. and Sandberg et al. included patients from the Northern part of Sweden and Stockholm [15, 16]. The patient group of four studies consisted of patients from the Children’s Hospital of Philadelphia [20–22, 27]. Three studies used the same registry to collect patient data [5, 19, 31]. Lastly, there was a high inter-study variability for measured outcomes; studies reported raw values, indexed values for height squared, or *Z*-scores, making absolute values hard to compare.

### Future and clinical applications

Future studies should be prospective to not only demonstrate associations but also investigate causes and include homogenous cohorts to minimalize confounders. Patients should be followed longitudinally, as this can provide a better insight into the development of the body composition over time and its role as a predictor for an adverse event. Importantly, means to improve the unfavorable body composition in patients with a Fontan circulation should be explored further.

## Conclusions

This systematic review shows that Fontan patients are predisposed to an unfavorable body composition, characterized by increased fat mass and decreased muscle mass accompanied by a normal BMI compared to the healthy population. Among others, unfavorable body composition was associated with decreased exercise capacity, cardiac function, and increased morbidity in patients with a Fontan circulation. Clinical awareness and appropriate management, including motivating patients with a Fontan circulation to maintain a healthy lifestyle, might lead to improved clinical outcomes.

### Supplementary Information

Below is the link to the electronic supplementary material.

Supplementary file1 (DOCX 62.5 KB)

## Data Availability

The data underlying this article will be shared on reasonable request to the corresponding author.
