# Supplementary material for: Body composition in patients with Fontan physiology: a systematic review
Source: Eur J Pediatr. 2023 Aug 5;182(10):4309–21. doi: 10.1007/s00431-023-05100-2 (PMC10587222; doi:10.1007/s00431-023-05100-2)
Supplement: Supplementary file 1 — Supplementary file1 (DOCX 62.5 KB) [file 431_2023_5100_MOESM1_ESM.docx]

Supplement 1. Searchterm per database

**Embase.com**

('congenital heart disease'/de OR 'congenital heart malformation'/exp OR 'heart right ventricle dysplasia'/de OR 'LEOPARD syndrome'/de OR 'systemic pulmonary shunt'/exp OR 'pulmonary valve atresia'/de OR 'tricuspid valve atresia'/de OR 'mitral valve atresia'/de OR 'aortic valve atresia'/de OR 'cardiomyopathy'/de OR (((congenital*) NEAR/3 (heart* OR cardiac*) NEAR/3 (disease* OR distress* OR malform* OR defect*)) OR (tetralog* NEAR/3 fallot*) OR aorto-ventricular-tunnel* OR cor-triatriatum* OR cyanotic-heart-disease* OR dextrocardia* OR Ebstein-anomaly* OR ectopia-cordis* OR Eisenmenger-complex* OR heart-left-right-shunt* OR heart-right-left-shunt* OR (sept* NEAR/3 defect*) OR cardiac-shunt* OR single-ventricle* OR hypoplastic-left-heart-syndrome* OR levocardia* OR myocardial-bridging* OR ((ductus* OR truncus*) NEAR/3 (arteriosus*)) OR velocardiofacial-syndrome* OR ventricular-noncompaction* OR ventriculoatrial-shunt* OR Fontan* OR systemic-pumonar*-shunt* OR ((transpos*) NEAR/3 (great* OR large*) NEAR/3 (vessel* OR arter*)) OR TGA OR heart-right-ventricle-double-outlet* OR (valv* NEAR/3 atresia*) OR cardiomyopath* OR (bidirectional* NEAR/3 cavopulmonar*-anastomos*) OR LEOPARD-syndrome*):ab,ti,kw) AND ('body composition'/exp OR 'skeletal muscle mass'/de OR 'skeletal muscle mass index'/de OR 'appendicular lean mass index'/de OR 'dual energy X ray absorptiometry'/de OR 'bioimpedance'/de OR 'bioimpedance spectroscopy'/de OR 'bioimpedance analysis'/de OR 'body composition analyzer'/exp OR 'fat free mass'/de OR (((body) NEAR/3 (compos* OR fat*)) OR (fat NEAR/3 distribut*) OR skeletal-muscle-mass* OR lean-mass* OR Skinfold-Thicknesses* OR fat-free-mass* OR Bioimpedance* OR Bio*-impedance* OR BIA OR BIVA OR Air-displacement-plethysmogra* OR BOD-POD OR PEA-POD OR Bodystat* OR InBody OR (dual NEXT/1 (energy OR emission*) NEXT/1 (X-ray* OR Roentgen OR Röntgen OR Xray)) OR DEXA OR DXA OR Dual-Photon-Absorpt*):ab,ti,kw) NOT [conference abstract]/lim NOT ((animal/exp OR animal*:de OR nonhuman/de) NOT ('human'/exp))

**Medline ALL Ovid 240**

(exp Heart Diseases/cn OR LEOPARD Syndrome/ OR Pulmonary Valve Stenosis / OR Tricuspid Atresia / OR Cardiomyopathies / OR (((congenital*) ADJ3 (heart* OR cardiac*) ADJ3 (disease* OR distress* OR malform* OR defect*)) OR (tetralog* ADJ3 fallot*) OR aorto-ventricular-tunnel* OR cor-triatriatum* OR cyanotic-heart-disease* OR dextrocardia* OR Ebstein-anomaly* OR ectopia-cordis* OR Eisenmenger-complex* OR heart-left-right-shunt* OR heart-right-left-shunt* OR (sept* ADJ3 defect*) OR cardiac-shunt* OR single-ventricle* OR hypoplastic-left-heart-syndrome* OR levocardia* OR myocardial-bridging* OR ((ductus* OR truncus*) ADJ3 (arteriosus*)) OR velocardiofacial-syndrome* OR ventricular-noncompaction* OR ventriculoatrial-shunt* OR Fontan* OR systemic-pumonar*-shunt* OR ((transpos*) ADJ3 (great* OR large*) ADJ3 (vessel* OR arter*)) OR TGA OR heart-right-ventricle-double-outlet* OR (valv* ADJ3 atresia*) OR cardiomyopath* OR (bidirectional* ADJ3 cavopulmonar*-anastomos*) OR LEOPARD-syndrome*).ab,ti,kw.) AND (exp Body Composition / OR Absorptiometry, Photon / OR (((body) ADJ3 (compos* OR fat*)) OR (fat ADJ3 distribut*) OR skeletal-muscle-mass* OR lean-mass* OR Skinfold-Thicknesses* OR fat-free-mass* OR Bioimpedance* OR Bio*-impedance* OR BIA OR BIVA OR Air-displacement-plethysmogra* OR BOD-POD OR PEA-POD OR Bodystat* OR InBody OR (dual ADJ (energy OR emission*) ADJ (X-ray* OR Roentgen OR Rontgen OR Xray)) OR DEXA OR DXA OR Dual-Photon-Absorpt*).ab,ti,kw.) NOT ((animal/ OR animal*:de OR nonhuman/) NOT (human/))

**Web of Science Core Collection 367**

TS=(((((congenital*) NEAR/2 (heart* OR cardiac*) NEAR/2 (disease* OR distress* OR malform* OR defect*)) OR (tetralog* NEAR/2 fallot*) OR aorto-ventricular-tunnel* OR cor-triatriatum* OR cyanotic-heart-disease* OR dextrocardia* OR Ebstein-anomaly* OR ectopia-cordis* OR Eisenmenger-complex* OR heart-left-right-shunt* OR heart-right-left-shunt* OR (sept* NEAR/2 defect*) OR cardiac-shunt* OR single-ventricle* OR hypoplastic-left-heart-syndrome* OR levocardia* OR myocardial-bridging* OR ((ductus* OR truncus*) NEAR/2 (arteriosus*)) OR velocardiofacial-syndrome* OR ventricular-noncompaction* OR ventriculoatrial-shunt* OR Fontan* OR systemic-pumonar*-shunt* OR ((transpos*) NEAR/2 (great* OR large*) NEAR/2 (vessel* OR arter*)) OR TGA OR heart-right-ventricle-double-outlet* OR (valv* NEAR/2 atresia*) OR cardiomyopath* OR (bidirectional* NEAR/2 cavopulmonar*-anastomos*) OR LEOPARD-syndrome*)) AND ((((body) NEAR/2 (compos* OR fat*)) OR (fat NEAR/2 distribut*) OR skeletal-muscle-mass* OR lean-mass* OR Skinfold-Thicknesses* OR fat-free-mass* OR Bioimpedance* OR Bio*-impedance* OR BIA OR BIVA OR Air-displacement-plethysmogra* OR BOD-POD OR PEA-POD OR Bodystat* OR InBody OR (dual NEAR/1 (energy OR emission*) NEAR/1 (X-ray* OR Roentgen OR Röntgen OR Xray)) OR DEXA OR DXA OR Dual-Photon-Absorpt*)) NOT ((animal* OR rat OR rats OR mouse OR mice OR murine OR dog OR dogs OR canine OR cat OR cats OR feline OR rabbit OR cow OR cows OR bovine OR rodent* OR sheep OR ovine OR pig OR swine OR porcine OR veterinar* OR chick* OR zebrafish* OR baboon* OR nonhuman* OR primate* OR cattle* OR goose OR geese OR duck OR macaque* OR avian* OR bird* OR fish*) NOT (human* OR patient* OR women OR woman OR men OR man)))

**Cochrane CENTRAL register of Trials**

((((congenital*) NEAR/3 (heart* OR cardiac*) NEAR/3 (disease* OR distress* OR malform* OR defect*)) OR (tetralog* NEAR/3 fallot*) OR aorto ventricular tunnel* OR cor triatriatum* OR cyanotic heart disease* OR dextrocardia* OR Ebstein anomaly* OR ectopia cordis* OR Eisenmenger complex* OR heart left right shunt* OR heart right left shunt* OR (sept* NEAR/3 defect*) OR cardiac shunt* OR single ventricle* OR hypoplastic left heart syndrome* OR levocardia* OR myocardial bridging* OR ((ductus* OR truncus*) NEAR/3 (arteriosus*)) OR velocardiofacial syndrome* OR ventricular noncompaction* OR ventriculoatrial shunt* OR Fontan* OR systemic pumonar* shunt* OR ((transpos*) NEAR/3 (great* OR large*) NEAR/3 (vessel* OR arter*)) OR TGA OR heart right ventricle double outlet* OR (valv* NEAR/3 atresia*) OR cardiomyopath* OR (bidirectional* NEAR/3 cavopulmonar* anastomos*) OR LEOPARD syndrome*):ab,ti,kw) AND ((((body) NEAR/3 (compos* OR fat*)) OR (fat NEAR/3 distribut*) OR skeletal muscle mass* OR lean mass* OR Skinfold Thicknesses* OR fat free mass* OR Bioimpedance* OR Bio* impedance* OR BIA OR BIVA OR Air displacement plethysmogra* OR BOD POD OR PEA POD OR Bodystat* OR InBody OR (dual NEXT/1 (energy OR emission*) NEXT/1 (X ray* OR Roentgen OR Rontgen OR Xray)) OR DEXA OR DXA OR Dual Photon Absorpt*):ab,ti,kw) NOT “conference abstract”:pt

Supplement 2. Quality Assessment per article

|  | Hansson et al.(15) | Sandberg et al. (16) | Sarafoglou et al.(24) | Tran et al.(5) | Ohuchi et al. (17) | Shiina et al.(25) | Cordina et al.(18) | Cao et al.(19) | Vaiknth et al.(22) | Possner et al.(26) | Powell et al.(23) | Avitabile et al.(20) | Avitabile et al.(21) | Chemello [32] | Tekerlek [30] | Avitabile (P) [27] | Pyykkönen [28] | Wadey [31] |
| --- | --- | --- | --- | --- | --- | --- | --- | --- | --- | --- | --- | --- | --- | --- | --- | --- | --- | --- |
| Abstract |  |  |  |  |  |  |  |  |  |  |  |  |  |  |  |  |  |  |
| Design in Title | x |  | x | x | x |  | x | x | x | x | x | x | x | x |  | x | x | x |
| Abstract |  |  |  |  |  |  |  |  |  |  |  |  |  |  |  |  |  |  |
| Introduction |  |  |  |  |  |  |  |  |  |  |  |  |  |  |  |  |  |  |
| Background |  |  |  |  |  |  |  |  |  |  |  |  |  |  |  |  |  |  |
| Objectives |  |  |  |  |  |  |  |  |  |  |  |  |  |  |  |  |  |  |
| Methods |  |  |  |  |  |  |  |  |  |  |  |  |  |  |  |  |  |  |
| Study design |  |  |  |  |  |  |  |  |  |  | x |  |  |  |  |  |  |  |
| Setting |  |  |  |  |  |  |  |  |  |  |  |  |  |  |  |  |  |  |
| Participants |  |  |  |  |  |  |  |  |  |  |  |  |  |  |  |  |  |  |
| Variables |  |  |  |  |  |  |  |  |  |  |  |  |  |  |  |  |  |  |
| Data source |  |  |  |  |  |  |  |  |  |  |  |  |  |  |  |  |  |  |
| Bias | x | x | x | x | x | x | x | x | x | x | x | x | x | x | x | x | x | x |
| Study size |  |  |  | x | x | x | x | x | x | x | x | x | x | x |  | x | x | x |
| Quantative variables |  |  |  |  |  |  |  |  |  |  |  |  |  |  |  |  |  |  |
| Statistical methods |  |  | x |  |  |  |  |  |  |  |  |  |  |  |  |  |  |  |
| Results |  |  |  |  |  |  |  |  |  |  |  |  |  |  |  |  |  |  |
| Participants |  |  |  |  |  |  |  |  |  |  |  |  |  |  |  |  |  |  |
| Descriptive data |  |  |  |  |  |  |  |  |  |  |  |  |  |  |  |  |  |  |
| Outcome data |  |  |  |  |  |  |  |  |  |  |  |  |  |  |  |  |  |  |
| Main results |  |  |  |  |  |  |  |  |  |  |  |  |  |  |  |  |  |  |
| Other analysis |  |  |  |  |  |  |  |  |  |  |  |  |  |  |  |  |  |  |
| Discussion |  |  |  |  |  |  |  |  |  |  |  |  |  |  |  |  |  |  |
| Key results |  |  |  |  |  |  |  |  |  |  |  |  |  |  |  |  |  |  |
| Limitations |  |  | x |  |  |  |  |  |  |  |  | x | x | x |  |  |  |  |
| Interpretation |  |  |  |  |  |  |  |  |  |  |  |  |  |  |  |  |  |  |
| generalizability |  | x | x | x | x | x | x | x |  |  |  |  |  |  |  |  |  |  |
| Funding |  |  |  |  |  |  | x |  |  |  |  |  |  |  |  |  |  |  |
| **Criteria (%)** | 91 | 91 | 77 | 82 | 82 | 86 | 77 | 83 | 87 | 87 | 83 | 83 | 83 | 83 | 96 | 86 | 86 | 86 |

X = points not given
